# Insulin and the kidneys: a contemporary view on the molecular basis

**DOI:** 10.3389/fneph.2023.1133352

**Published:** 2023-08-03

**Authors:** Rodrigo Daza-Arnedo, Jorge Rico-Fontalvo, Gustavo Aroca-Martínez, Tomás Rodríguez-Yanez, María Cristina Martínez-Ávila, Amilkar Almanza-Hurtado, María Cardona-Blanco, Carlos Henao-Velásquez, Jorge Fernández-Franco, Mario Unigarro-Palacios, Carolina Osorio-Restrepo, Isabella Uparella-Gulfo

**Affiliations:** ^1^ Department of Nephrology, Colombian Association of Nephrology, Cartagena, Colombia; ^2^ Faculty of Medicine, Universidad Simón Bolívar, Barranquilla, Colombia; ^3^ Department of Internal Medicine, Universidad de Cartagena, Cartagena, Colombia; ^4^ Department of Internal Medicine, Universidad El Bosque, Bogotá, Colombia; ^5^ Department of Internal Medicine, Endocrinology Fellowship, Fundación Universitaria de Ciencias de la Salud—Hospital San José, Bogotá, Colombia; ^6^ Specialist in Clinical Pharmacology, Universidad de la Sabana, Bogotá, Colombia; ^7^ Universidad del Sinu, Cartagena, Colombia

**Keywords:** insulin, diabetic kidney disease, kidney, diabetes mellitus, homeostasis

## Abstract

Insulin is a hormone that is composed of 51 amino acids and structurally organized as a hexamer comprising three heterodimers. Insulin is the central hormone involved in the control of glucose and lipid metabolism, aiding in processes such as body homeostasis and cell growth. Insulin is synthesized as a large preprohormone and has a leader sequence or signal peptide that appears to be responsible for transport to the endoplasmic reticulum membranes. The interaction of insulin with the kidneys is a dynamic and multicenter process, as it acts in multiple sites throughout the nephron. Insulin acts on a range of tissues, from the glomerulus to the renal tubule, by modulating different functions such as glomerular filtration, gluconeogenesis, natriuresis, glucose uptake, regulation of ion transport, and the prevention of apoptosis. On the other hand, there is sufficient evidence showing the insulin receptor’s involvement in renal functions and its responsibility for the regulation of glucose homeostasis, which enables us to understand its contribution to the insulin resistance phenomenon and its association with the progression of diabetic kidney disease.

## 1 Introduction

Insulin is a hormone composed of 51 amino acids, structurally organized as a hexamer comprising three heterodimers (1). Insulin is the central hormone involved in the control of glucose and lipid metabolism, maintaining processes such as cell growth and body homeostasis (2). Insulin was isolated and purified in Toronto between 1921 and 1922, and this milestone can be attributed to Drs Frederick Banting and Charles Best (1). Since its discovery, there have been intensified scientific efforts to optimize the function of this hormone, which has also increased the study of other hormones, such as glucagon, resulting in an ostensible improvement in the survival of patients with diabetes mellitus (DM). Diabetes is a chronic, multifactorial, polygenic, potentially reversible disease characterized by chronic hyperglycemia in relation to decreased insulin production, decreased insulin sensitivity by the target tissues (i.e., adipocyte, muscle, or liver), or a combination of both. Uncontrolled DM can lead to microvascular and macrovascular complications. Diabetic kidney disease (DKD) (3) can occur in up to 30%–40% of patients with type 1 and 2 diabetes mellitus (2), of which approximately 20% are already on dialytic therapy, increasing the percentage of patients in end-stage renal disease and with cardiovascular complications (3).

Previously, it was thought that insulin alone was present only in the beta cells of the pancreas; however, recent evidence shows that it is also present at low concentrations in the central nervous system.

Glucose metabolism is stimulated by food intake, which simultaneously leads to increased beta-cell insulin production and decreased glucagon secretion by the alpha cells; these processes maintain serum glucose levels at a normal range. Following secretion, insulin circulates systemically and is distributed in hepatocytes, promoting glucose storage in the form of glycogen. Skeletal muscle and adipocytes are the primary target organs of circulating insulin, and are also responsible for peripheral glucose uptake, reducing plasma glucose concentrations. Similar to other protein hormones, insulin triggers glucose uptake, protein synthesis through skeletal muscle, glycogenesis, and lipogenesis through the tyrosine kinase receptor pathway (1).

The insulin receptor is found in plasma membranes that act enzymatically to transmit ATP phosphates to tyrosine residues, with action on target proteins found intracellularly, followed by the binding of insulin to the alpha subunit, phosphorylating the beta subunit by activating the catalytic receptor. The activated receptor further results in the phosphorylation of several intracellular proteins regulating the metabolic activity of insulin, cell growth, cell differentiation, and the expression of genes related to these processes (3).

## 2 Kidney–insulin relationship

The kidney, in conjunction with the liver, works to maintain basal insulin levels. Normally, the kidney eliminates between 6 and 8 units of insulin by two mechanisms (1): post-glomerular secretion, and (2) glomerular filtration (4). These processes will be discussed throughout this article.

On the other hand, insulin acts at multiple sites along the nephron, from the glomerulus to the renal tubule, to regulate functions such as glomerular filtration and gluconeogenesis, which are determinants of blood glucose levels. Insulin also influences renal sodium transport, which has an impact on the water status of patients (5).

## 3 Insulin synthesis

Insulin is synthesized as a large preprohormone and has a leader sequence or signal peptide that appears to be responsible for transport to the endoplasmic reticulum membranes, where this peptide signal is hydrolyzed by a peptidase, forming proinsulin. The hydrolysis of proinsulin via proconvertases 1, 2, and 3 and carboxypeptidase results in the formation of the two insulin chains. Equimolar amounts of C-peptide are also formed. Insulin and C-peptide are stored in equimolar amounts in the secretion granules. When a suitable stimulus arrives, the granules are fused to the plasma membrane by releasing equimolar amounts of insulin and C-peptide into the circulation. This is the reason that C-peptide levels reflect endogenous insulin production (6).

## 4 Insulin secretion

Insulin is a peptide hormone secreted from the beta cells of the pancreas. The human pancreas contains between one and two million pancreatic islets, called Langerhans islets, which respond to variations in blood glucose concentration, and, subsequently, are where insulin secretion occurs (6). This organ receives approximately 10% of the cardiac output (7). Although insulin secretion is controlled by a complex series of nerve (neurotransmitters), hormonal (gastrointestinal hormones), and nutritional signals, glucose is considered the first regulatory signal for insulin secretion. The minimal glucose concentration for insulin secretion is 80–100 mg/dL, which corresponds to fasting plasma glucose levels; the maximum response is obtained with glucose levels of 300–500 mg/dL (8).

Insulin secretion is a sequential process primarily occurring in pancreatic beta cells, the purpose of which is to fuse secretory granules with the plasma membrane and to exocytosize granules. Insulin is primarily secreted in response to glucose; however, hormones, such as melatonin, estrogens, leptin, growth hormone, and glucagon-like peptide 1 (GLP-1), also regulate the process. The increase of intracellular Ca^2+^ is known to be the primary signal for insulin secretion; however, other mechanisms, such as those dependent on cAMP signaling, are known to be of relevance to insulin secretion (x1).

For a better understanding of the physiology of insulin secretion, it has been organized into a series of sequential stages (6):

Insulin secretion is presented as a pivotal response to the stimulation of glucose ingress into the beta cells of the pancreas through GLUT-2 facilitated diffusion. Once inside the beta cells, glucose is phosphorylated to glucose-6-phosphate, as presented below (8). This glucose interaction with GLUT2 occurs in both hepatocytes and pancreatic beta cells that express the GLUT2 transporter and are located on the plasma membrane. This receptor has a low affinity for glucose, which is critical for glucose uptake in hyperglycemia states (8).

### 4.1 Glucose phosphorylation

The enzyme glycokinase, present in the liver and in the pancreatic beta cells, acts as a substrate for glucose, generating its phosphorylation and transformation into glucose-6-phosphate, which follows the glycolytic route, so that the product (pyruvate) is degraded to acetyl CoA and can enter the Krebs cycle in the mitochondria.

Mitochondrial ATP production and ATP-dependent closure of potassium (K^+^) channels: ATP production from the Krebs Cycle increases the ATP/ADP ratio, resulting in ATP-dependent K-channel closure.

### 4.2 Cell depolarization

ATP-dependent K^+^-channel closure increases intracellular K^+^ by generating a voltage change that causes the depolarization of the beta-cell membrane. (X2).

Aperture of voltage-dependent calcium (Ca^2+^) channels: once the cell membrane is depolarized by voltage changes, the Ca^2+^ channels are opened, which allows calcium into the cell; this phase allows the passage from proinsulin to insulin, which is packed in vesicles to be released into the bloodstream through exocytosis.

### 4.3 Exocytosis

Exocytosis is a complex process in which the SNARE [soluble *N*-ethylmaleimide-sensitive fusion factor (NSF)-binding protein receptor] proteins are involved in an ATP-dependent process; here, it is included with the v-SNARE (soluble NSF attachment protein receptor) protein or synaptobrevin in the secretory vesicle membrane, t-SNARE or syntaxin in the plasma membrane, and SNAP25, which forms a four-square, capable of disrupting the lipid bilayer. Once this process is established, the outer layers of the membranes fuse, and the fusion with the inner layers is then complete, allowing insulin to escape. The NSF protein then separates the proteins involved in the process.

Insulin secretion follows a biphasic pattern: a first phase, which is transient, followed by a second phase, which is sustained. In humans, when plasma glucose concentrations are ≈ 7 mM, the first-phase insulin secretion peaks at a maximum of 1.4 nmol/min. The first phase lasts ≈ 10 min and is then followed by the second phase, with a secretion rate of ≈ 0.4 nmol/minute (X2).

It appears that insulin granules can be classified into different functional groups [X3]. A small fraction of the fast-releasing granules (1%), called the readily releasable group (RRP), contribute to the rapid release of glucose-induced insulin [X4]. The remaining granules (99%) belong to the backup group. When the RRP is depleted, it is refilled from the booking group. The backup group granules must undergo preparatory reactions before becoming RRP granules (9).

## 5 Insulin receptor

Insulin acts on a transmembrane receptor consisting of two alpha and beta subunits. It belongs to the growth-factor receptor family and is a glycoprotein with a tyrosine kinase effect (10). When in contact with insulin, the beta subunit undergoes a conformational change in its catalytic domain, which is activated and phosphorylated; this is the starting point for the cell signaling phenomenon (11).

## 6 Insulin signaling

**Figure 1:**
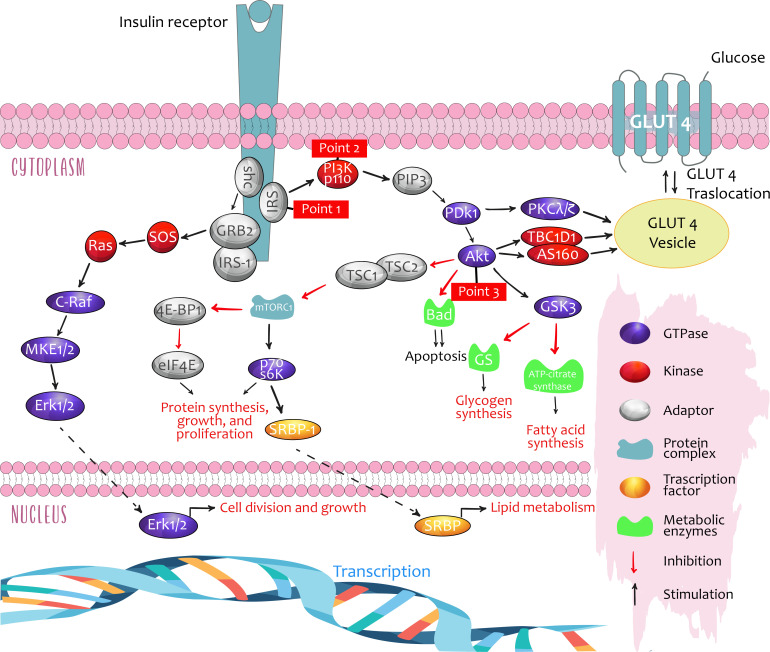
Critical points of regulation in insulin signalling. Taken from: Role of Insulin in Health and Disease: An Update, *Int. J. Mol. Sci.* 2021, 22, 6403.

Insulin signaling activates three critical regulatory points, through which it acts to perform its metabolic actions in its target tissues: muscle, adipose tissue, and liver (**Figure 1**).

*Point 1 contains the receptor for insulin substrate isoforms (IRSs) 1–4.
**Point 2 is the enzyme phosphatidylinositol 3-kinase (PI3K), which consists of a regulatory subunit (p85 or p55) and a catalytic subunit (p110).
***Point 3 contains three isoforms of Akt (rho–alpha serine/threonine kinase family).

These three signaling points regulate lipid and glucose metabolism, in addition to cell growth and differentiation. Furthermore, they run in parallel with the Ras-MAPK pathway (mitogen-activated proteins kinases such as MEK, MAPK/KRE), with some crossover (7, 12).

Insulin, by binding to its receptor, allows the splicing and activation of multiple signaling molecules, especially IRS, PI3K, and Akt, which may act as mediators with different physiological effects, depending on the tissue:

1. In muscles, glucose uptake and metabolism are increased by translocation of the insulin-regulated glucose transport protein (GLUT4), furthermore promoting protein synthesis and suppressing atrophy through regulation of the genes *FoxO* and *MTORC1*. Evidence from experimental studies in murine models showing that exposure to impoverished water in deuterium (a stable hydrogen isotope) stimulated GLUT4 translocation in the presence of insulin, leading to a decrease in glycemia, was recently published. The data demonstrate a potentially positive effect of metabolic regulation on insulin resistance (5).
2. In adipose tissue, glucose uptake and triglyceride production are increased from glycerol phosphate 3 (lipogenesis) while suppressing lipolysis.
3. In the liver, insulin promotes lipogenesis and counteracts the actions of glucagon, thereby suppressing glucose production.

## 7 Biological effects of insulin on nephrons

**Figure 2:**
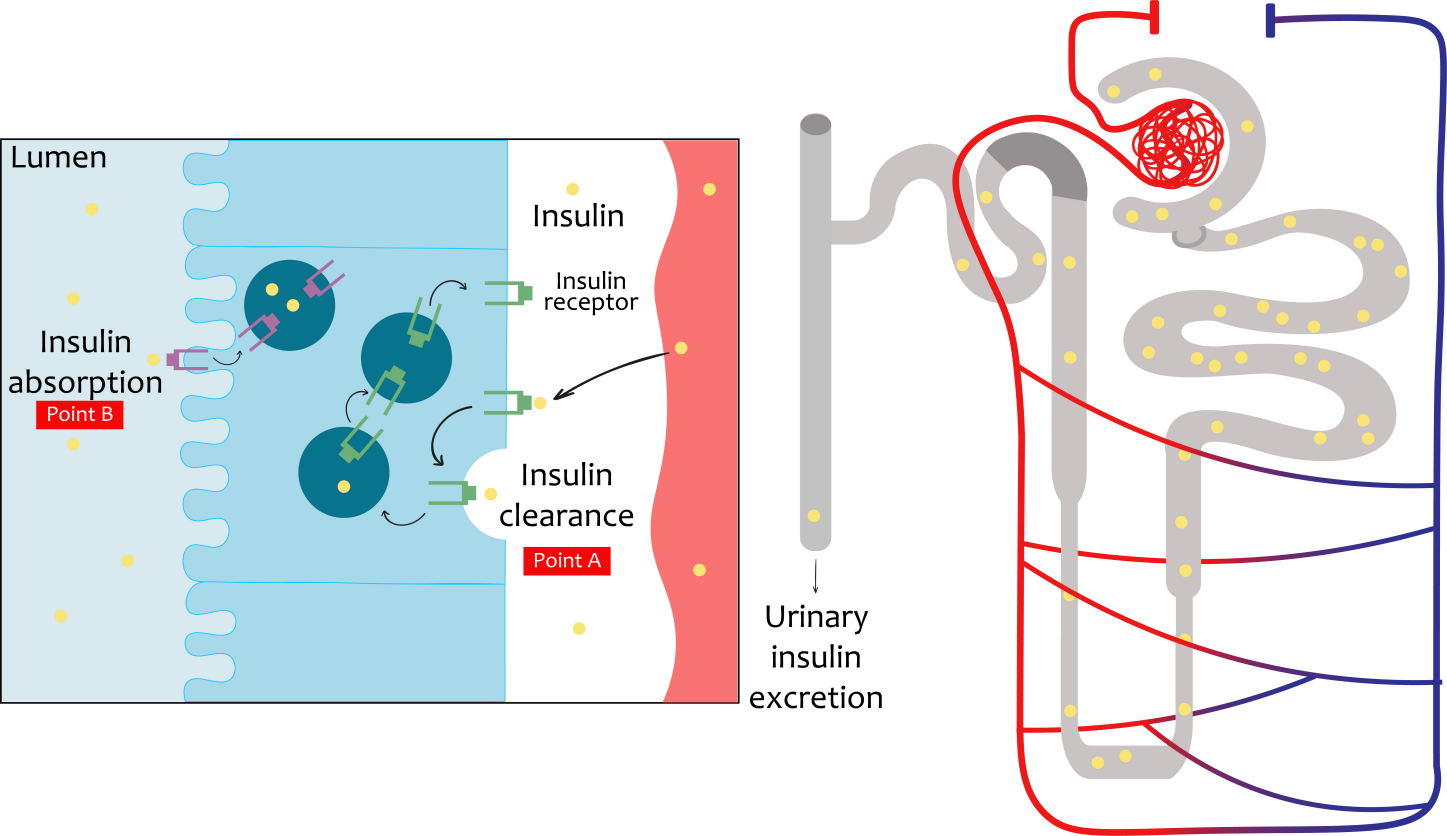
Insulin travel in the renal system. The insulin, being a small molecule, will be filtered completely down into the glomerular system into the proximal tubule. In the proximal tubule cells, the totality of the filtered insulin will be absorbed into the luminal membrane. Under normal conditions, only a small percentage will be excreted in urine. Beyond glomerular filtration, insulin also ascends from the perivenous capillaries. In the proximal tubule cells the mechanisms of insulin elimination are represented. Insulin receptor increased (INSR) levels and consequent increased insulin uptake in the basolateral membrane are also represented.

The initial evidence of the actions of insulin in the kidneys was published in the 1950s (6). Insulin may act on all renal cell types, including mesangial cells, podocytes, and tubular epithelial cells. Both insulin receptor isoforms are widely expressed in the kidney (2). The interaction of insulin with the kidney is a dynamic and multicenter process, acting on tissues ranging from the glomerulus (13) to the renal tubule (14), modulating different functions such as glomerular filtration, gluconeogenesis, natriuresis, glucose uptake, the regulation of ion transport, and prevention of apoptosis. The kidney also works in tandem with the liver to maintain balance in insulin levels. Due to this, it eliminates, on average, between 6 and 8 units of insulin per day through two routes (**Figure 2**). These pathways are intended for post-glomerular secretion (insulin signaling) and glomerular filtration (nurture homoeostasis) (4, 15).

## 8 Glomerular effects of insulin

**Figure 3:**
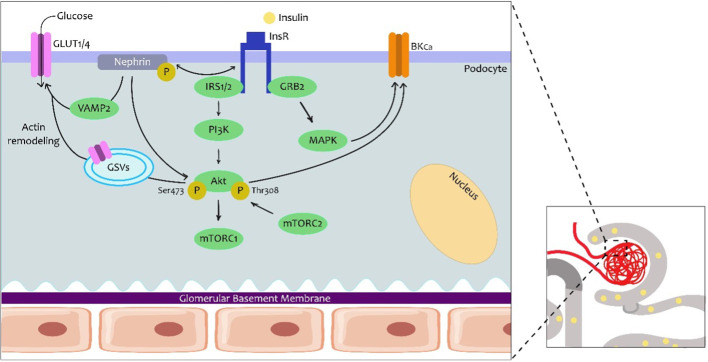
Insulin signalling in the podocytes. Podocytes are the first cells to interact with insulin in the nephron and express several proteins from the canonical insulin signalling pathway. However, here it is known that the podocyte-specific nephrine protein plays a role in the trafficking of glucose transporters (GLUT1 or GLUT4) to the podocyte membrane and consequently promotes glucose uptake. The traffic seems to involve Vamp2 and actin remodeling. In the other arm of insulin signalling, an effect on Ca2+-activated K+ channels of high conductance (BKCa) is also important for the maintenance of podocyte integrity and adequate glomerular filtration. GRB2, protein 2 attached to growth factor receptor; GSV, GLUT storage vesicle; VAMP2, vesicle-associated membrane protein 2.

Podocytes are the major constituent cell of the glomerulus, with their long digitiform projections toward the glomerular capillaries on the glomerular basal membrane (GBM). These cells have intercellular junctions that form filtration barriers to help maintain normal renal function. When there is damage to the podocytes, they lose their disposition, resulting in a reduction in barrier function. In fact, one of the characteristics of diabetic nephropathy is the loss of podocytes with consequent albuminuria. In the last few decades, insulin function and its relation to podocytes have remained prominent (**Figure 3**) (15, 16).

Podocytes express proteins from the canonical insulin signaling pathway, namely the INSR, and both the IRS1 and the IRS2, with the IRS2 having the highest prevalence (14). In podocytes, insulin not only facilitates glucose uptake via GLUT4 but also via glucose transporter 1 (GLUT1). Insulin promotes the translocation of GLUT4 to the membrane by activation of the PI3K–AKT2–PkB pathway, resulting in remodeling of the cortical actin of the cytoskeleton (17) A major agonist in podocyte physiology is nephrin, a podocyte-specific protein, which is responsible for maintaining the integrity of the filtration barrier. In fact, nephrin mutations are involved in severe nephrotic syndromes (18). Nephrin appears to play a more prominent role in the trafficking of GLUT4 and GLUT1 by interacting with Vamp2 and with insulin-stimulated actin remodeling (19).

The effects of insulin on podocyte function add to the increasing evidence that demonstrates that it plays a predominant role in the metabolism and function of these cells (17). Initially, it was demonstrated that insulin increased the activation of the protein kinase G type I alpha (PKGIα) subunit, which is related to podocyte dysfunction. In addition, a relationship has been established between this subunit and oxidative stress, rearrangement of the actin and changes in permeability that favor albumin filtration. Insulin also stimulates the expression of the pore-forming subunits of Ca^2+^-activated K^+^ channels or BKCa (Slo1 proteins) in mouse podocyte models. A more recent study demonstrated the role of insulin in increasing the permeability of the filtration barrier through mobilization of BKCa-channels through PKGI in cultured rat podocytes (20). This molecular mechanism could contribute to the lesion of podocytes, proteinuria, and albuminuria, which is typical of DKD.

Recently, advances have been made in the understanding of the alterations in the cytoskeleton of podocytes and calcium metabolism as a critical element in the pathogenesis of renal disease, the alterations in glomerular filtration, and the occurrence of albuminuria. The increased expression and/or podocyte-specific activity of Orai1, mediated by insulin activity, alters glomerular filtration through alterations in the concentration of Ca^2+^ in podocytes, as has been demonstrated in animal models and cell cultures. In addition, these alterations in calcium metabolism in podocytes, mediated by alterations in insulin receptor tyrosine kinase (RTK) signaling, link actin remodeling, and, therefore, the podocyte cytoskeleton with the deletion of the podocyte process and resulting in proteinuria. Consequently, alterations in intracellular calcium concentration and metabolism would be another element to be considered in the pathophysiology of the development of insulin-mediated proteinuric renal disease (21).

## 9 Tubular effects of insulin

**Figure 4:**
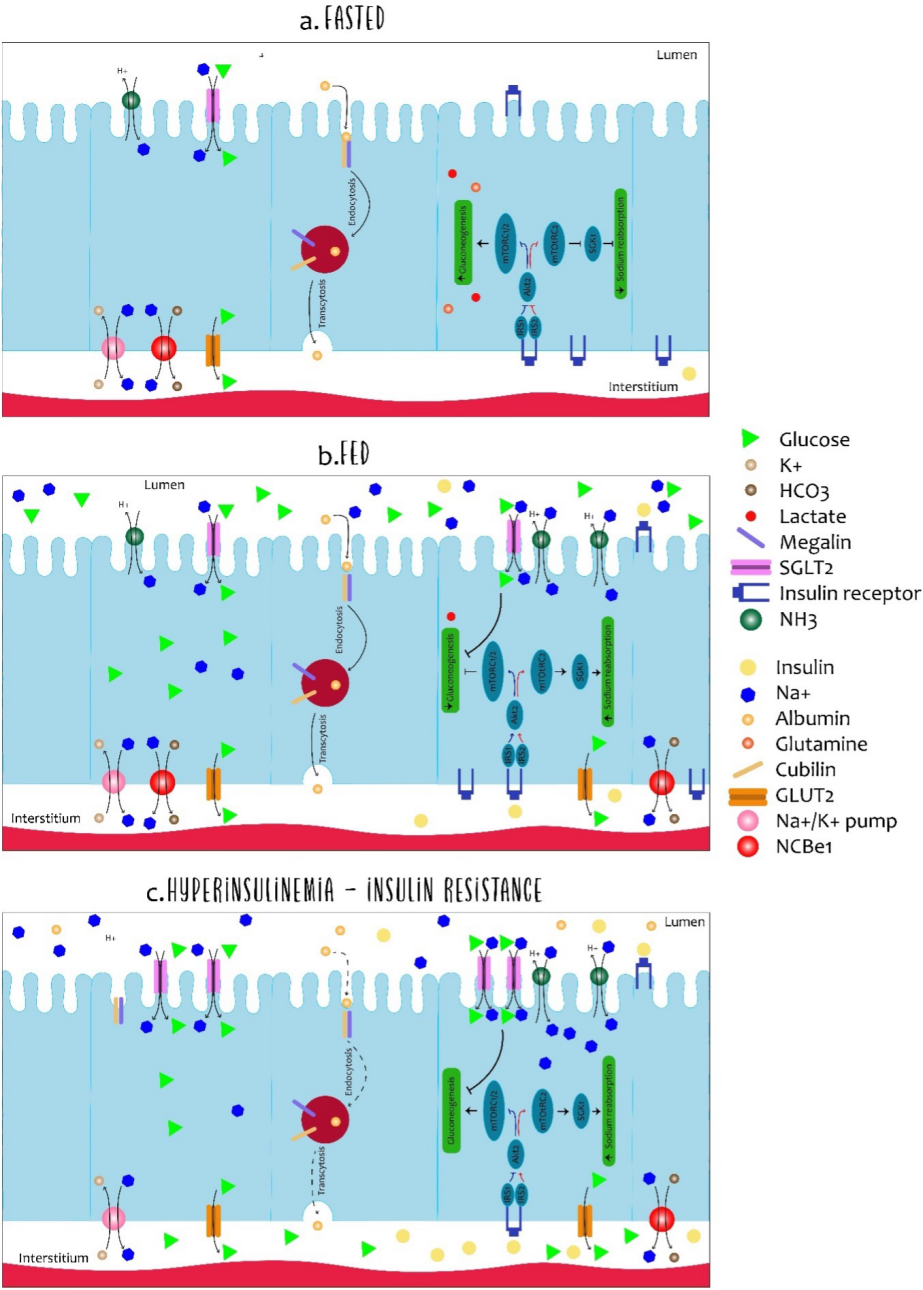
Dynamics of cells in the proximal tubule in fasted, fed and insulin-resistant states. The cells of the proximal tubule are subject to different microenvirons (light and interstitium) and the regulation of the absorption and resorption of molecules is complex. Although all described processes occur in each cell of the proximal tubule simultaneously, each specific process is illustrated in a different cell. In the fasting state **(A)**, low levels of insulin allow expression of glyconeogenic enzymes while sodium reabsorption is regulated in the low. The expression of glucose transporter 2 (GLUT2) in the basolateral membrane is primarily associated with glucose output and not with uptake. Furthermore, albumin absorption is performed by megalin and cubilin in the luminal membrane and transcytosis allows albumin to deviate back into the body. In the fed state **(B)**, the increased availability of insulin and glucose promotes dramatic changes in the dynamics of the proximal tubule. For insulin, luminal uptake is mostly associated with degradation and basolateral to signalling activation. Insulin receptor activation (INSR) down-regulates gluconeogenesis and increases sodium reabsorption by different proteins such as the Na-H type 3 exchanger (NHE-3) and sodium-glucose transporter protein 2 (SGLT2). Along with sodium, SGLT2 also co-transports glucose from light. Finally, hyperinsulinemia is related to disturbances of the proximal tubule cells in many aspects **(C)**. As in many other organs, desensitization of insulin signalling is associated with inefficient inhibition of gluconeogenesis contributing to the maintenance of elevated glucose levels. Podocyte-level disturbances increase albumin filtration and overload the luminal capacity of resorption. Such impairment in albumin resorption culminates in albuminuria, commonly observed in hyperinsulinemic states.

In the renal tubule, insulin has several functions: metabolism, electrolyte regulation, acid–base balance, and the absorption of filtered substances. However, the exact mechanisms by which insulin performs these different functions are not fully known. However, it seems that at least some of them are mediated by the INSR (22). The insulin receptor is known to be present throughout the nephron (23); however, its binding capacity to insulin from the intratubular space to the tubular cells in the basolateral membrane is different. **Figure 4** summarizes the insulin signaling in the renal tubule with respect to its actions on both gluconeogenesis and sodium reabsorption. In addition, the actions of insulin through the INSRs are believed to be different in the proximal and distal regions of the nephron (15).

The kidney plays an important role in gluconeogenesis along with the liver and intestine, which are primarily produced in proximal tubular cells (PTs), essentially from lactate and glutamine, as illustrated in **Figure 4**. PT cells, on the other hand, do not require the use of glucose, they obtain energy mainly from the oxidation of fatty acids. Insulin is responsible for regulating gluconeogenesis in PT cells, and, thereby, providing fluctuations of body energy requirements. In the fasting state, the kidney contributes 40% to the glyconeogenesis process, but being in the state of absorption, this contribution falls to 20%, demonstrating that insulin directly inhibits gluconeogenesis in PT cells in a manner isolated by the interaction of the IRS1/AKT2/mTORC1/2.

In the fasting state, suppression of insulin signaling increases FoxO1 activity, which increases the expression of glyconeogenic genes, such as *PEPCK* and glucose-6-phosphatase. At the same time, glucose absorption through SGLT2 decreases in the luminal membrane by decreasing the NADH/NADC ratio. On the other hand, the postprandial state increases insulin levels to favor glucose reabsorption and the suppression of gluconeogenesis by decreasing the gene expression favoring this process (15).

## 10 Renal degradation

Insulin cannot be detected at the plasma level 30 minutes after its release from the pancreas, and its plasma half-life is approximately 6 minutes (24). Furthermore, hepatic clearance clears 50% of insulin after the first step, and 25% after the second step. The hormone is also slowly internalized by most cells, including myoblasts and adipocytes, where it targets the lysosome for degradation, with this being a mechanism for ending its action. The degradation of insulin is only administered in a circulating fraction, which passes through the liver, and, subsequently, reaches the kidney; here, its fate is divided into three steps:

(1) Filtration into the glomerulus. Insulin is rapidly resorbed into the renal epithelial cells; this resorption involves a saturable binding of low affinity and high capacity on the brush border membrane, where it has been shown not to be via the IR insulin receptor but possibly via receptors such as megalin, which is part of the lipoprotein receptor family, and cubilin. Insulin is internalized through a retro-endocytosis pathway where it dissociates with its binding site and is transported to the lysosomes to end its degradation (25).

(2) An equal amount of insulin also enters the renal tubular cells of a counter luminal localization reaching the tubular capillaries, especially in the contoured tubule, here the insulin receptors (IRs) of the epithelial cells are attached to the insulin to be transported intracellularly. Furthermore, these are important signaling pathways as they detect hormones to stimulate functions such as sodium, phosphate, and glucose reabsorption. These two renal mechanisms of insulin internalization have been proposed to be responsible for eliminating up to 6–8 units of insulin per day, which is equivalent to 25% of secreted insulin from the pancreas, or 50% of circulating insulin, although this is believed to be an overestimation. However, renal insulin clearance may explain the surprising fact that patients with type 1 diabetes and renal failure may end up with a reduced need for applied insulin (7).

(3) The majority of insulin is internalized and degraded by the above-mentioned pathways. A small fraction is reabsorbed into the renal circulation through retro-endocytosis. Obviously, altered renal clearance prolongs its blood half-life, showing the importance of these processes in the half-life of insulin in the blood circulation (7).

## 11 Insulin and renal resistance

The deterioration of insulin action results in its resistance in the tissues of the target organs. The kidney is an organ involved in the clearance of insulin; however, whether or not it develops resistance to it is still debated. The regulation of insulin receptor (IR) expression and function is very well studied in major tissues, such as skeletal muscle, liver, and adipose. The role of IRs in the kidney has been clarified recently and continues to be studied. Considering its wide distribution in nephron segments, it has been possible to identify insulin in animal models, in which IRs are diminished in insulin resistance states, thereby allowing the determination of the degree of importance in normal and pathological states related to insulin resistance (23, 26).

## 12 Insulin receptor and its renal expression

The insulin receptor was first isolated in 1972 from hepatocyte and adipocyte cell membranes. Subsequently, it was shown to be a homodimer bound to a disulfide (12, 27). Its DNA was separated in 1985, revealing that the alpha chain extends to the N-terminal of the beta chain, separated by a proteolytic cleavage site (12). The role of IR in nephrons has been studied since the last decade; however, some of its functionality has emerged recently. Animal models with rats have shown that their high affinity at attachment sites in the contoured tubule (i.e., in their proximal and distal part) is lowered in the collector tubule (i.e., in their medullary and cortical part) (28). Localization of these receptors in animal studies demonstrated that their distribution pattern in the nephron is unique and does not overlap the IGF-1 receptor (6).

In the kidney, the reabsorption of Na^+^ is administered through several segments of the nephron. Insulin has an antinatriuretic effect that increases resorption of Na^+^, and, furthermore, regulates different Na^+^ channels including Na^+^/H^+^ type 3, a sodium bicarbonate cotransporter, Na^+^-K^+^-ATPase Na^+^/Cl^−^, a co-transporter, and Na epithelial channels. The interaction of insulin–IR and renal absorption of sodium remains an investigational focus for understanding the connection between insulin resistance and arterial hypertension. On the other hand, hyperinsulinemia affects the renal flow mediated by the nitric oxide (NO) concentration. Under normal conditions, insulin has a vasodilator effect, which is related to NO production (nitric oxidation). Another recent study documents for the first time, the activation of eNOS (endothelial nitric synthetase oxide) and the generation of insulin-induced NO, in the collector tubules cells in the internal medullary region (29, 30).

The renin–angiotensin–aldosterone system (RAS) is another mechanism for controlling systemic blood pressure and for hydroelectrolytic equilibrium. The classical RAS pathway leads to sodium reabsorption, vasoconstriction, and increased blood pressure. It has also been established that angiotensin II inhibits insulin-mediated activation of PI3K. In addition, an interrelationship between insulin resistance and the RAS pathway has been identified, although its precise mechanism has not been fully clarified (31, 32). Ang II had previously been shown to induce phosphorylation from IRS1, which is a key IR substrate, forming Ser^616^ and Ser^312^, which is responsible for its inactivation and inhibition in the insulin/PI3K signaling pathway (23, 33).

## 13 Kidney–glucose relationship

The plasma glucose concentration is determined by five fundamental pillars (1): intestinal glucose absorption (2), hepatic glycogenolysis (3), renal glucose reabsorption (4), renal glucose excretion, and (5) both renal and hepatic gluconeogenesis (34). Thus, the kidney intervenes in three of these five mechanisms, showing that this organ plays a preponderant role.

With respect to the renal reabsorption of glucose, in normal conditions, this mechanism allows all the glucose that was previously filtered to be rescued, resulting in glucose-free urine, or urine with minimal amounts of glucose. It should be remembered that approximately 90% of glucose is reabsorbed in the S1 segment of the proximal tubule, where SGLT2 and GLUT2 transporters are located, and the remaining 10% is reabsorbed in the S3 segment, where SGLT1 and GLUT1 predominate (35).

On the side of renal gluconeogenesis, this process consists of the synthesis of glucose from precursors, such as lactate, glutamine, alanine, and glycerol, by the cells of the renal cortex, which have glucose 6-phosphatase enzymatic activity, contributing to approximately 20% of the body’s glucose release (36).

## 14 Renal function in systemic glucose homeostasis

The role of IR signaling is of vital importance in the regulation of blood glucose levels; however, knowledge of its action in the kidney is limited. These receptors are known to influence this homeostasis through various mechanisms such as glucose reabsorption, glucose uptake and gluconeogenesis. Patients with diabetes have abnormalities in these regulatory mechanisms. Some studies have previously suggested that renal epithelial cells double glucose uptake in response to insulin stimulation by the translocation of GLUT receptors to the plasma membrane (mainly type 1 and 4), accentuating the effect of insulin on renal gluconeogenesis and blood glucose levels (23, 37). These studies have highlighted the importance and magnitude of renal glucose production, in addition to having a higher sensitivity to the renal glucose release compared with the liver. A marked decrease in IR expression has been observed in experimental studies with fat-fed rats. Another study showed that the selective elimination of IR in the proximal tubule caused hyperglycemia despite normal insulin sensitivity. In addition, increased activity and higher expression of glucose-6-phosphatase mRNA were observed, suggesting the involvement of IR in the expression of key glyconeogenic genes (*G6Pase–PEPCK*). All these studies suggest that impaired insulin sensitivity in the proximal tubule may fully affect glucose homeostasis through increased gluconeogenesis through transcriptional induction of renal glyconeogenic enzymes. However, the mechanism by which IR signaling leads to transcription of glyconeogenic genes in the proximal tubule is still being studied (26, 38). In conclusion, studies in animal models may imply that altered and decreased renal insulin signaling, mainly of the IR, may increase gluconeogenesis in the insulin resistance scenario, leading to other harmful effects (39).

Another aspect that relates to the interaction of insulin–IR in the kidneys is the presence of proteinuria, such as albumin, which represents it as a distinct feature of diabetic kidney disease (40). Impaired tubular function contributes to proteinuria. Albumin resorption is administered by means of endocytic, megalin, and cubilin receptors that are highly expressed in the apical membrane of the proximal tubule cells. There is evidence to suggest that insulin could potentially play a role in albumin absorption. Studies in animal models in which diabetes was induced, showed dysregulation in the expression of pSer473-Akt in the epithelial cells of the proximal tubule, which was also accompanied by a decrease in the expression of megaline and cubicin, which established an insulin and proteinuria ratio. On the other hand, insulin treatment in hyperglycemic mice increased the expression of megaline. These reports establish a causal relationship in the pathogenesis of albuminuria (23, 41).

## 15 Renal RI and proteinuria

The presence of proteins is the main characteristic of diabetic nephropathy ERD (40). Although glomerular dysfunction is an established cause of proteinuria, impaired tubular function also contributes to albuminuria in ERD. Albumin is normally reabsorbed by the proximal tubule (PT) cells through receptor-mediated endocytosis by megalin, and aculin, which are highly expressed in the apical membrane of PT cells. The existing evidence suggests that, in addition to other factors, insulin could play a role in the absorption of albumin by PT cells in diabetic and non-diabetic conditions. The recovery of albumin from ultrafiltration by PT cells is crucial for renal homeostasis. A cohort study in non-diabetic individuals (relation between insulin sensitivity and RISC cardiovascular disease) proposed the existence of a causal relationship between insulin resistance and albuminuria. The RISC study demonstrated that the reduction in insulin sensitivity mediated by euglycemic hyperinsulinemia is related to an increased risk of albuminuria in a healthy cohort (42).

## 16 Insulin inflammation and resistance

Inflammation is another notable mechanism that contributes to the occurrence of insulin resistance. Chronic mediators of inflammation, such as tumor necrosis factor alpha (TNF-α), interleukin 6 (IL-6) and liraglutide, have shown increased levels in patients with chronic kidney disease. The binding of insulin to its IR receptor is preserved, but the translation signals after binding seem to be impaired, with this causality inhibiting the degradation of IRIS1 by the ubiquitin complex, which reduces Akt phosphorylation, resulting in abnormal metabolism of hemostatic glucose and lipids (26, 43). Hyperglycemia correlates with the development of inflammation, a situation that is precipitated by different mechanisms in the patient with chronic kidney disease and related to immune processes. Hyperglycemia results in the activation of different pathways beyond the classical hemodynamic and metabolic axes, such as that of inflammatory cascades (2). For example, one of the mechanisms is the presence of mainly type 4 Toll-like receptors (TLRs), of which there is an overexpression at the renal tubular level, and are associated with interstitial infiltration by the macrophages in patients with type 2 diabetes, which correlates with the progression of chronic kidney disease and decreased glomerular filtration rate (2, 44).

Insulin resistance is involved in the development and progression of DKD. It contributes to the development of glomerular hypertension and hyperfiltration, conditions frequently observed in the early stages of DKD, at which time metabolic and hemodynamic alterations leading to disease progression tend to predominate (45, 46). In both T1D and T2D, insulin resistance contributes to increased salt sensitivity, increasing blood pressure, albuminuria, and a decline in renal function. These components contribute to the hemodynamic alterations typical of DKD, the description of which escapes the objectives of this article (47). In addition, insulin resistance is a common condition in patients with CKD, independent of the presence of DM, obesity, fasting, or age, and is therefore a preponderant factor in the development and progression of renal disease (48, 49).

During these early stages of DKD, insulin resistance is associated with poor glycemic control and upregulation of the Na^+^/glucose transporter SGLT2. It contributes to an increase in tubular resorption of salt, specifically sodium (Na^+^), and also the secondary worsening or loss of hyperfiltration by the physiological action of the feedback pathways of tubule–glomerular systems (50, 51). However, multiple factors, independent of the presence of DM, have been associated with the presence of insulin resistance. These include metabolic acidosis, accumulation of uremic toxins, proinflammatory states, vitamin D deficiency, intestinal microbiota disturbances, and decreased adiponectin (49). Insulin resistance is generally a factor in the progression of CKD and its incidence increases with the evolutionary stages of renal disease.

## 17 Renal glyconeogenesis and insulin

A decrease in renal gluconeogenesis promotes a new distribution of endogenous glucose production, between glucose-producing organs during fasting. This is characterized by an increase in intestinal glyconeogenesis, leading to the storage of glycogen in the liver. Decreasing the level of circulating vitamin D affects renal gluconeogenesis deficiency and could play a key role in this process by lowering the inhibitory role of insulin in intestinal gluconeogenesis, triggering a neuronal communication between the gut, brain, and liver. These data shed light on the interference that takes place between glucose-producing organs, which could occur in situations of impaired renal glucose production, such as chronic kidney disease (52).

Consequently, insulin downregulates renal glyconeogenesis, just as it does in the liver. It is estimated that the kidneys account for approximately 15%–20% of the total daily glucose production under normal conditions. In the advanced chronic kidney disease scenario, these functions are reduced, which contributes to the risk of hypoglycemic events occurring, which are inherent to the patient experience with renal pathology (49).

## 18 Insulin, liver, and kidney interaction

The kidney is also considered a gluconeogenic organ, in contrast to what was previously thought and participates in approximately 20% of glucose production. The remaining 80% is generated by the liver, and the kidney contains the enzymes necessary for this process (36, 53). Gluconeogenesis is inhibited by insulin, through its action in both the kidneys and liver, the cells of the renal cortex generate glucose, and this glucose is used in the renal medulla, maintaining a renal corticomedullary homeostasis (53). In the diabetic patient with hyperglycemia, the frequency of glucose generation increases, and at the same time, serum insulin levels increase, indicating the presence of insulin resistance in both organs (54). There is also the participation of other substances, such as epinephrine, which stimulates the production of glucose in the kidneys only, whereas glucagon acts in only the liver (36).

The role of the kidney is fundamental in this interaction: in healthy adults, it can reabsorb approximately 375 mg/minute, which is much less than the amount of glucose filtered, and this can be understood as the mechanism of the absence of glucosuria in healthy individuals (55, 56).

The behavior of the diabetic patient exceeds these thresholds of normality, losing the balance between filtration and renal reabsorption of glucose. In DM2, there is a change in both the maximum transport of glucose reabsorption and the threshold for glucosuria (57). When sodium concentrations decrease in the cytoplasm, sodium re-enters the renal cell together with glucose. In addition, given that this mechanism would be associated with an increase in body sodium, this mechanism is related to arterial hypertension (53).

## 19 Urinary proteome

The term proteome refers to the complete set of proteins expressed by an organism (58). This term can also be used to refer to the group of specific proteins produced and expressed by a specific organ or tissue (59). On the other hand, proteomics is a science that involves developing technologies that investigate the structure and function of the set of proteins that make up the proteome (58, 59). In the case of the renal proteome, these are the sets of proteins expressed by the kidney, which are produced by specific renal cells (59).

Under normal conditions, urine contains small amounts of low- and medium-molecular-weight proteins; however, in pathological situations, such as diabetic kidney disease, there are alterations in the type, quantity, and quality of these proteins, which has aroused medical interest in recent years as a tool for the early detection and risk stratification of patients with chronic kidney disease (60).

The presence in urine of collagen fragments, α1-microglobulin, β2-microglobulin (β2MG), α1 antitrypsin (A1AT), and uromodulin, among other peptides, has shown that they could be promising biomarkers to detect individuals with T2DM who are at high risk of developing CKD (61).

## 20 Collagen fragments

The presence of collagen fragments in urine could be an early predictor of diabetic kidney disease in patients with normoalbuminuric DM1 and DM2 between 3 and 5 years before the onset of significant albuminuria (61). Similarly, collagen α-1 and α-5 were also increased in individuals with DM2, diabetic nephropathy, and CKD not associated with diabetes (62). It has also been suggested that collagen is a more specific biomarker than urinary albumin (63).

## 21 Uromodulin

Uromodulin is present in the CKD273 classifier, and its expression has been documented as being decreased in patients with DM2, non-diabetic CKD, and diabetic nephropathy (62).

## 22 α1 antitrypsin

The protein α1 antitrypsin is also part of CKD273, and a significant increase has been seen in patients with DM1 and DM2 (64).

## 23 Haptoglobin

There is evidence to support haptoglobin being a good urine biomarker for predicting the early deterioration of renal function (65).

## 24 β2-microglobulin

Increased β2MG and ubiquitin excretion has been found to be higher in DM patients, with higher levels being present in those who have established diabetic nephropathy (66).

## 25 α1-microglobulin

Its increase in urine has been documented in patients with diabetic nephropathy, both in patients with DM1 and DM2 (67).

## 26 UbA52

UbA52 is a ubiquitin ribosomal fusion protein, the selective excretion of which has been observed in DM2 patients with significant albuminuria (68).

Urinary protein in diabetic kidney disease would allow early diagnosis and delay the onset of renal complications.

The presence of collagen fragments, α1-microglobulin, β2MG, A1AT, and uromodulin appear to be promising biomarkers for detecting patients with DM2 at high risk of developing chronic kidney disease. The presence of high levels of collagen fragments in the urine of type 1 and 2 diabetic patients can predict their likelihood of developing diabetic kidney disease between 3 and 5 years before the onset of macroalbuminuria (59).

Uromodulin, another biomarker present in CKD273, was also downregulated in cases of nephropathy. Other proteins that are related to diabetic kidney disease have also been found in the urine of diabetic patients, such as increased 1 beta glycoprotein, zinc 2 glycoprotein, 2 HS glycoprotein, vitamin D binding protein, calgranulin B, A1AT, and hemopexin.

The urinary proteome has the potential to be used in clinical practice. Type 1 collagen has been identified as one of the most important markers and promises to be of great utility in daily clinical practice, and other markers, such as uromodulin and lucin-1, are no less important (59).

## 27 Conclusion

A full analysis of the physiological pathway of insulin at body level and the relevance of physiology and its renal function was performed through mediated and non-mediated mechanisms of the insulin receptor, with its different biological and non-biological effects on different nephron segments being observed. Not only does it exert its principal action on organs, such as the liver, muscle, and fat tissue, but also on renal epithelial cells, which are gaining increasing research attention. Studies in animal models, especially in rats, have shown that there is an altered insulin receptor at the renal level and that this may be related to renal processes, both physiological and pathological. The insulin receptor has shown sufficient evidence of its renal function and its responsibility for the regulation of glucose homeostasis by gluconeogenesis, lipolysis, and sodium homeostasis, which makes it possible to understand its contribution to the insulin resistance phenomenon and its association with the progression of diabetic kidney disease. This study’s findings show that insulin has great potential to act as a therapeutic target and an intervention measure for adequate control of the disease.

## Author contributions

JR-F, RD-A, and GA-M: protocol preparation, data recruitment, outcome analysis, and article writing. TR-Y, AA-H, MM-A, CH-V, MU-P, and JF-F: conception and design of the research, analysis of results, writing of the article, and analysis of the conclusions. CO-R and IUG: literature search, data recruitment, outcome analysis, and article review. MC-B: article review and conclusions analysis. All authors contributed to the article and approved the submitted version.
